# An Adolescent Patient With Idiopathic Pulmonary Arterial Hypertension Weaned Off Intravenous Epoprostenol Following Treatment With Selexipag: A Case Report

**DOI:** 10.3389/fped.2022.909595

**Published:** 2022-06-17

**Authors:** Ayako Chida-Nagai, Takao Tsujioka, Daisuke Sasaki, Gaku Izumi, Hirokuni Yamazawa, Atsuhito Takeda

**Affiliations:** Department of Pediatrics, Hokkaido University Hospital, Sapporo, Japan

**Keywords:** pulmonary arterial hypertension, epoprostenol, drug transition, adolescence, selexipag

## Abstract

Idiopathic pulmonary arterial hypertension (PAH) is a rare, progressive disease affecting the pulmonary arteries. Epoprostenol, a synthetic prostaglandin analog, is the most potent pharmacological treatment modality used in patients with PAH. However, it requires continuous intravenous infusion, which negatively impacts the patient’s quality of life and frequently results in complications, such as catheter-related bloodstream infection. We weaned an adolescent female patient off epoprostenol by gradually introducing oral selexipag over a sustained period, following many years of continuous intravenous epoprostenol use alone. Oral selexipag might have an efficacy comparable to epoprostenol in young patients with PAH.

## Introduction

An adolescent female patient with idiopathic pulmonary arterial hypertension (PAH) was being treated with epoprostenol; however, the PAH remained severe. When she was 14 years of age, oral selexipag was added to her regimen of epoprostenol, and her condition improved. At the age of 17 years, the patient was weaned off epoprostenol. This method of switching and weaning off has the potential to improve the quality of life of young patients with idiopathic PAH.

## Case Presentation

The patient was born at 41 weeks fetal age by normal vaginal delivery; her birth weight was 3,200 g, and there was no history of neonatal asphyxia. She was apparentlyasymptomatic until the age of 5 years, when, after crying, her face went pale and her level of consciousness transiently decreased. A heart murmur and cough were noted during a medical check-up performed immediately after the event, and she was admitted to our hospital for further investigation. After a thorough examination, including ultrasound cardiography and cardiac catheterization (Table 1), she was diagnosed with idiopathic PAH. She had no family history of PAH. Genetic testing revealed no pathological variants in *BMPR2, ACVRL1, ENG, SMAD9, CAV1, KCNK3*, and *EIF2AK4*, which are the genes responsible for heritable PAH. Although beraprost, sildenafil, and bosentan were administered, her dyspnoea continued to worsen, and she developed pulmonary hypertensive (PH) crisis. Three months after the initial diagnosis, she underwent cardiac catheterization to facilitate the initiation of epoprostenol therapy. When intravenous anesthesia was administered prior to the insertion of the cardiac catheter, she suffered an acute PH crisis again and went into cardiopulmonary arrest. Two days after she was weaned off cardiopulmonary support, a computed tomography scan of the head revealed extensive cerebral infarction. Hypoxic encephalopathy was confirmed, and tracheostomy, central venous (CV) catheter implantation, gastrostomy, as well as Nissen fundoplication were subsequently performed. Thereafter, the patient was transferred to a long-term care facility, where she was followed up with increasing doses of epoprostenol, bosentan, and sildenafil. At the age of 6 years, beraprost was discontinued.

**TABLE 1:** Cardiac catheterization result at 5 years of age.

PA pressure (mmHg)	85/58 (77)
PA wedge pressure (mmHg)	8
LV (mmHg)	97/EDP11
RV (mmHg)	86/EDP18
RpI (Woods units⋅m^2^)	40.7
C.I. (l/min⋅m^2^)	2.2

*EDP, end-diastolic pressure.*

**FIGURE 1:**
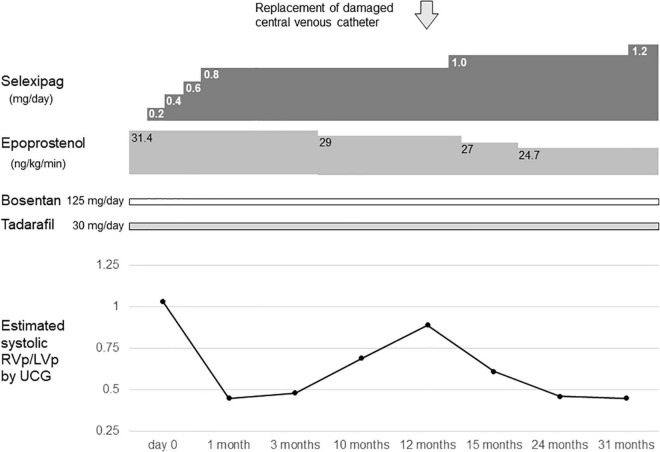
Timeline of selexipag initiation at the age of 14–16 years. Day 0 indicates the day on which selexipag was initiated. RVp, right ventricular pressure; LVp, left ventricular pressure; UCG, ultrasoundcardiography.

**FIGURE 2:**
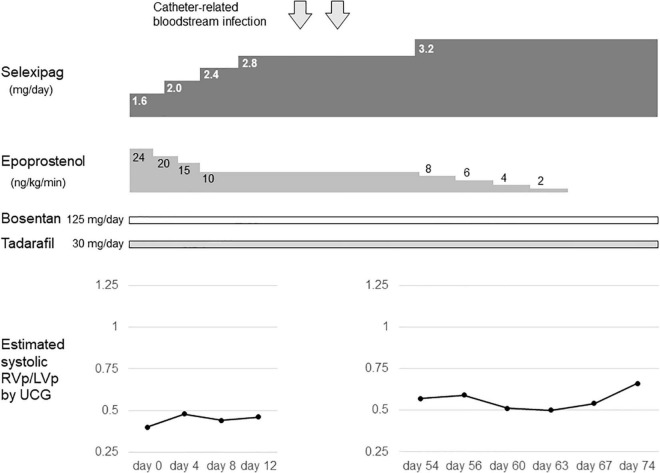
The drug transition timeline at the age of 17 years. Day 0 indicates the day on which the dose of selexipag was increased to 1.6 mg/day. RVp, right ventricular pressure; LVp, left ventricular pressure; UCG, ultrasoundcardiography.

At 11 years of age, she had a catheter-related bloodstream infection and was treated by antibiotics. At 13 years of age, she was switched from sildenafil to tadalafil, owing to tadalafil’s longer half-life in comparison to sildenafil and its once-daily-dose effectiveness. Even after increasing the dose of epoprostenol during subsequent outpatient visits, the estimated systolic right ventricular pressure on ultrasound cardiography was comparable to the systolic left ventricular pressure. Therefore, when she was 14 years of age, she was admitted to our hospital, and treatment with selexipag was initiated. After titration of selexipag to 0.8 mg/day, ultrasound cardiography showed that the estimated systolic right ventricular pressure improved to approximately 50% of the systolic left ventricular pressure (Figure 1). At the age of 15 years, her CV catheter broke and had to be replaced. At 17 years of age, we increased the dose of selexipag to 2.8 mg/day and decreased the dose of epoprostenol from 31 to 10 ng/kg/min (Figure 2). Shortly thereafter, there were two more instances of catheter-related bloodstream infection; thus, we decided to begin the process of weaning her off epoprostenol. After increasing the dose of selexipag to the maximum daily dose (3.2 mg/day), epoprostenol was tapered and discontinued over a period of 2 weeks. After the discontinuation of epoprostenol, the estimated right ventricular pressure as perultrasound cardiography was approximately 60% of the left ventricular pressure (Figure 2). The patient had hypoxic encephalopathy and was, thus, unable to complain of headache, jaw pain, or other symptoms. Therefore, it was difficult to determine whether there were any adverse side effects from increasing the dose of selexipag. However, we proceeded with the drug transition while monitoring her facial expressions, pulse rate, blood pressure, and sleep status, and determined that there were no severe adverse reactions. Brain natriuretic peptide levels in the blood remained below detectable ranges throughout the clinical course.

## Discussion

Pulmonary vasodilators used to treat PAH are classified into three types based on their pathway of action: the endothelin pathway, the nitric oxide pathway, and the prostacyclin pathway (1). Epoprostenol was the first prostanoid to be approved as a PAH-specific drug, and many patients have benefited from its use (2). However, due to its short half-life, it requires continuous intravenous infusion. This makes its use restrictive in daily life and, as seen in this case, can lead to complications, such as CV catheter breakage and catheter-related bloodstream infections. Thus, the development of an effective oral PH treatment comparable to epoprostenol is the need of the hour.

Selexipag is a relatively new oral prostacyclin receptor agonist, which was approved for use in Japan in 2016. Its safety and efficacy have been established in pediatric patients with PAH (3, 4).

To our knowledge, our patient is the first adolescent with PAH who was successfully weaned off epoprostenol with the addition of selexipag over a prolonged duration of time. There have been several recent reports regarding this drug transition in adults with PAH (5, 6). We took an extended period of time to carefully transition this patient from her regimen of epoprostenol to stand-alone treatment with selexipag, given that she was a child and suffered from hypoxic encephalopathy. The long-term course of this patient, who has only recently been transitioned to stand-alone selexipag, will need to be closely monitored in the future.

A concern in this case report is that no cardiac catheterization was performed before or after the transition from epoprostenol to selexipag. This is because cardiac catheterization in this patient was prohibited, following the cardiopulmonary arrest that occurred during cardiac catheterization at the age of 5 years; however, frequent ultrasound cardiography was used to safely monitor drug transition.

Herein, the estimated systemic right ventricular pressure on ultrasound cardiography decreased from the time of initial selexipag induction (0.8 mg/day), indicating a sensitive response. It is expected that there will be variability in the response of patients to selexipag. Further investigation is warranted in larger populations, besides the two available pediatric studies (3, 4) to assess the efficacy across a more diverse sample size.

In conclusion, we demonstrated that the transition from epoprostenol to selexipag can be safely performed in an adolescent patient with PAH. The extended duration of transition helps better analyze potential complications and drug response. Individualized assessment of cases is critical to identify patients with PAH in whom this transition is feasible.

## Data Availability Statement

The raw data supporting the conclusions of this article will be made available by the authors, without undue reservation.

## Ethics Statement

Ethical review and approval were not required for the study on human participants in accordance with the local legislation and institutional requirements. Written informed consent to participate in this study was provided by the patient’s legal guardian. Written informed consent was obtained from the minor’ legal guardian for the publication of any potentially identifiable images or data included in this article.

## Author Contributions

AC-N drafted the manuscript. All authors listed have contributed to the clinical care and management of the patient.

## Conflict of Interest

The authors declare that the research was conducted in the absence of any commercial or financial relationships that could be construed as a potential conflict of interest.

## Publisher’s Note

All claims expressed in this article are solely those of the authors and do not necessarily represent those of their affiliated organizations, or those of the publisher, the editors and the reviewers. Any product that may be evaluated in this article, or claim that may be made by its manufacturer, is not guaranteed or endorsed by the publisher.
